# Participant-reported recovery-related outcomes during routine community mental health support: a CHIME-informed longitudinal evaluation

**DOI:** 10.3389/fpubh.2026.1909643

**Published:** 2026-09-23

**Authors:** Olga Isabel Fernández-Rodríguez, Cristina García-Bravo, María Fernández-Blanco, Paloma Guillén-Rogel, Raquel Martínez-Sinovas, Beatriz Fernández-Díez, Alicia Cal-Herrera

**Affiliations:** 1 INPRO (Research in Occupational Participation and Performance), Department of Health Sciences, European University Miguel de Cervantes, Valladolid, Spain; 2 Department of Physical Therapy, Occupational Therapy, Rehabilitation and Physical Medicine, Rey Juan Carlos University, Alcorcón, Spain

**Keywords:** CHIME framework, community mental health, empowerment, occupational performance, personal recovery, psychosocial rehabilitation

## Abstract

**Background:**

Recovery-oriented community mental health services seek to support personal recovery, everyday participation, and person-centered care, but these outcomes may not improve in parallel. Objective: To examine longitudinal changes in personal recovery, perceived person-centered care, occupational performance, and satisfaction with performance among adults receiving routine community support in the Spanish public social services system. The Connectedness, Hope and optimism about the future, Identity, Meaning in life, and Empowerment framework informed outcome evaluation and interpretation.

**Methods:**

A longitudinal pre–post evaluation without a comparison group included 159 adults receiving routine community mental health support. Participant-reported outcomes were assessed at baseline and follow-up using the 24-item Recovery Assessment Scale, the Spanish version of the Client-Centred Rehabilitation Questionnaire, and the Canadian Occupational Performance Measure. Separate linear mixed models were fitted for each primary outcome, adjusting for sex, age, primary diagnostic group, primary support program domain, primary support intensity, secondary support receipt, and time-by-sex interaction. Sensitivity analyses examined alternative model specifications.

**Results:**

Occupational performance increased from baseline to follow-up (adjusted mean change 0.73, 95% confidence interval 0.46–0.99; Holm-adjusted *p*-value <0.001), as did satisfaction with performance (0.86, 95% confidence interval 0.59–1.13; Holm-adjusted *p*-value <0.001). No statistically significant longitudinal change was detected in personal recovery (0.85, 95% confidence interval −1.81 to 3.52; Holm-adjusted *p*-value = 0.529) or perceived person-centered care (0.11, 95% confidence interval −0.05 to 0.26; Holm-adjusted *p*-value = 0.361). No primary outcome showed evidence of differential change by sex. Sensitivity analyses yielded the same substantive conclusions.

**Conclusion:**

Complementary recovery-related outcomes may follow different trajectories during routine community support and should not be treated as interchangeable indicators of recovery. These findings highlight the value of multidimensional participant-reported outcome monitoring alongside individualized planning to inform the ongoing review and adaptation of community support to changing needs, priorities, and life goals. Systematic multidimensional outcome monitoring may help determine whether planned supports actually remain aligned with changing needs, priorities, and life goals, and may strengthen the ongoing individualization and quality improvement of community mental health services.

## 1 Background

Over recent decades, mental health care has increasingly moved beyond models focused primarily on symptom reduction toward a broader understanding of personal recovery. Anthony conceptualized recovery as a deeply personal process involving changes in attitudes, values, feelings, goals, skills, and roles through which a person can develop a satisfying, hopeful, and meaningful life despite the limitations associated with mental illness (1). From this perspective, recovery is not defined solely by clinical remission but also encompasses how people reconstruct their identity, develop expectations for the future, pursue valued goals, assume meaningful roles, and participate in community life. The relevance of this understanding has subsequently been supported across different cultural and diagnostic contexts (2, 3).

Within this conceptual development, Connectedness, Hope and optimism about the future, Identity, Meaning in life, and Empowerment (CHIME) has become one of the most influential frameworks for understanding personal recovery. Based on a systematic review and narrative synthesis, Leamy et al. identified five interrelated recovery processes: Connectedness, Hope and optimism about the future, Identity, Meaning in life, and Empowerment (4). This framework conceptualizes recovery as a multidimensional process defined from the perspective of the person rather than as a single clinical outcome. These processes are interrelated but are not equivalent. Evidence further distinguishes functional, social, and existential recovery outcomes (5), while personal recovery shows only partial associations with clinical recovery and functioning (6). Together, these findings caution against assuming that different recovery-related outcomes necessarily reflect the same underlying change.

Contemporary recovery-oriented practice is also embedded within a broader framework of human rights, citizenship, and social participation. The United Nations Convention on the Rights of Persons with Disabilities recognizes principles including autonomy, respect for individual will and preferences, access to the support required to exercise legal capacity, and the right to live independently and participate fully in the community (7). The World Health Organization (WHO) has translated these principles into mental health policy and practice through its QualityRights initiative and guidance on community mental health services, promoting person-centered, recovery-oriented, and rights-based models connected with essential areas of everyday life, including housing, education, employment, and social protection (8, 9). From a citizenship perspective, recovery is also connected with access to the rights, responsibilities, roles, resources, and relationships associated with full membership in society, together with belonging and recognition within the community (10). Autonomy, supported decision-making, social inclusion, and meaningful participation therefore constitute central elements of community responses that are recovery-oriented and respectful of people’s life projects (7–10).

These principles have direct implications for the organization of community mental health services. Person-centered, recovery-oriented care requires more than simply providing services in community settings, as it involves organizing support in relation to each person’s life history, needs, preferences, strengths, and valued goals while enabling meaningful participation in decisions concerning care and everyday life (8, 11). Translating these principles into practice also depends on the organizational and structural conditions within which services operate. Implementation evidence identifies service flexibility, relationships, available resources, organizational and policy commitment, and the capacity to support diverse personal recovery goals as relevant factors in the implementation of recovery-oriented services (12). In Spain, advances, opportunities, and barriers have similarly been identified in translating recovery-oriented, person-centered, and rights-based principles into public mental health policies and routine service practice (13).

The empirical evidence illustrates both the potential and the complexity of recovery-oriented approaches. In a systematic review including 309 studies, Winsper et al. found that recovery-oriented interventions were associated with functional, social, and existential recovery outcomes through a range of processes underpinned by four common mechanisms: providing information and skills, promoting a working alliance, role modeling recovery, and increasing choice (5). Harvey et al., in a systematic review of community-based models for people with persistent and complex mental health needs, identified a range of models associated with clinical, functional, or personal recovery outcomes, but none warranted classification as best practice. Their review also highlighted the centrality of service-user goals and priorities in emerging models supporting personal and functional recovery and emphasized the need for models to evolve and adapt to heterogeneous needs (14).

Recent evidence from Spain further illustrates the complexity of these processes. Sampietro et al. evaluated a structured recovery-planning workshop implemented in community mental health services using a non-randomized controlled design with 152 participants (15). Outcomes related to personal recovery, Empowerment, and Hope differed when participants’ initial stage of recovery was taken into account, suggesting that recovery-related outcomes may also depend on where individuals are situated within their recovery process (15). These findings are consistent with broader evidence showing that personal recovery is related to, but not equivalent to, clinical and functional recovery and that different recovery-related outcomes may reflect distinct processes and mechanisms (5, 6, 14).

This multidimensionality creates an important challenge for outcome evaluation. No single instrument can be assumed to represent the full range of processes encompassed by CHIME. Vogel et al. identified content corresponding to all five CHIME processes within the Recovery Assessment Scale, although representation was uneven and particularly strong for Empowerment (16). Penas et al. similarly highlighted limitations in the correspondence between different recovery measures, including the Recovery Assessment Scale, and the complete five-process CHIME framework (17). More broadly, Felix et al. identified 25 self-report measures of personal recovery and found that no measure had sufficiently strong evidence across all measurement properties examined (18). These findings support the use of complementary outcomes when recovery is approached as a multidimensional process (16–18).

Existing evidence therefore encompasses a wide range of recovery-oriented interventions and community-based models, with substantial heterogeneity in the models evaluated, populations, mechanisms, and outcomes (5, 14). Recent evidence from Spain has also examined a specifically structured recovery-planning intervention (15). Together, this evidence leaves an important question for routine community services: whether complementary outcomes reflecting personal recovery, person-defined occupational performance and participation priorities, and the experience of person-centered care show similar or differentiated trajectories during usual community support. Examining these outcomes together may provide additional insight into recovery processes under real-world service conditions. This question is particularly relevant when individualized planning based on people’s needs, preferences, and goals must be translated into support within existing organizational structures and service resources (12, 14).

The multidimensional nature of CHIME provides a useful conceptual framework for addressing this question because it enables personal, relational, and everyday-life-related outcomes to be interpreted together without reducing recovery to a single measure (4, 5). Its selection was also informed by the conceptual congruence between the recovery processes represented within CHIME and key characteristics of the community support model evaluated, including individualized planning based on life history, identified needs, preferences, and personal goals, promotion of autonomy, and support for participation in community life (4, 8, 11, 12, 14).

Against this background, the primary aim of the present study was to examine, using CHIME as a conceptual framework for outcome evaluation and interpretation, longitudinal changes in personal recovery, perceived person-centered care, occupational performance, and satisfaction with performance among adults receiving routine community support within the Spanish public social services system. We hypothesized that favorable changes would be observed between baseline and follow-up across these complementary recovery-related outcomes. In addition, differences in outcomes according to sex, primary diagnostic group, and support characteristics were examined. Exploratory analyses further assessed whether longitudinal trajectories differed by sex and whether they varied jointly according to sex and primary diagnostic group.

## 2 Materials and methods

This study is part of a broader mixed-methods research project registered in the Open Science Framework (OSF; https://doi.org/10.17605/OSF.IO/6BVKD). A longitudinal pre–post evaluation without a comparison group was conducted to examine changes in recovery-related outcomes during a predefined period of routine community mental health support. The study was reported in accordance with the Transparent Reporting of Evaluations with Nonrandomized Designs statement (19).

### 2.1 Participants and recruitment

El Puente Salud Mental (Valladolid, Spain), a nonprofit community-based mental health association in Spain, which provides psychosocial and recovery-oriented support services for adults with mental disorders. The collaborating organization functions as a community-based resource within a broader regional mental health care network. Individuals may access this type of community resource on their own initiative or through referral from the regional public health service; referrals may occur, for example, after hospital-based treatment has been completed and ongoing community support is required. Other public mental health services are available according to clinical need, including day hospital care for individuals who continue to require support for active symptoms. No concurrent comparison group was included because the study was designed as a single-group longitudinal evaluation embedded in routine implementation of the regional support framework rather than as a comparative effectiveness study. Withholding or delaying routine support for research purposes was not considered appropriate in this service context; alternative observational comparison strategies were not part of the original evaluation design.

The sample consisted of individuals recruited through purposive sampling based on the following inclusion criteria: aged 18 or older; a confirmed diagnosis of a mental disorder established by a psychiatrist and documented in the clinical information provided by the collaborating organization; identified support needs; and voluntary consent to participate in the study. Exclusion criteria included current substance intoxication or abuse that could interfere with participation, and the presence of active psychotic symptoms that might hinder comprehension during the interview (e.g., hallucinations, delusions, or disorganized thinking).

At the time of study planning, no sufficiently comparable previous studies were identified that could provide a directly transferable expected longitudinal effect size for the present support model, population, setting, and outcome framework. Therefore, sample size was estimated *a priori* to ensure adequate precision in describing the target service population rather than on the basis of a prespecified longitudinal effect size. Assuming a 95% confidence level (*α* = 0.05) and a conservative expected proportion of 0.50 in the absence of prior data, the prespecified sample-size target was 157 participants. Under this conventional precision-based calculation, a sample of 157 corresponds to an approximate nominal precision of ±8 percentage points for a population proportion of 0.50.

### 2.2 Community support program

The community support program evaluated in this study was delivered as part of routine community mental health support within the Spanish public social services system by professionals from the collaborating organization, who were independent of the research team. Support was provided within a person-centered planning framework based on the life-history support model established by the regional public administration. Each participant took part in an individual interview with a professional from the collaborating organization. Information derived from the person’s life history, current needs, preferences, and personal goals was used to develop an individualized support plan in accordance with the standardized procedures and service portfolio of the regional social services system. Each participant had a designated professional of reference assigned by the collaborating organization. Support contacts were delivered individually and were organized in accordance with each participant’s individualized support plan. Each participant received at least one structured support contact per week, tailored to their identified needs and personal goals. Their content varied according to the participant’s identified needs, preferences, personal goals, and assigned support program and could address areas such as vocational preparation and employment, community living and housing, social reintegration, leisure and community participation, or personal assistance. The research team was not involved in assigning the professional of reference or in organizing or delivering these routine support contacts. Support was delivered by a multidisciplinary community team comprising professionals from psychology, social work, occupational therapy, and related disciplines. These support contacts did not follow a standardized session-by-session research protocol; rather, their content was individualized within the routine service framework.

The evaluation coincided with the implementation of an updated regional framework for organizing and planning community support promoted by the Junta de Castilla y León, the regional public administration. The updated framework formed part of a broader regional shift toward improving support through a person-centered model organized around the individual’s life project. It emphasized identified needs, preferences and personal goals, autonomy and community participation, and continuity of support across changing circumstances. Within the service evaluated here, implementation of this framework involved developing or revising individualized support plans and could lead to continuation, modification, or reorganization of supports already being received rather than necessarily initiating a new service. All participants were observed for a period of at least 3 months before follow-up; however, community support itself was not limited to the evaluation period and continued thereafter as part of routine service provision.

The community support model was not designed or delivered using CHIME as an intervention framework or service-delivery protocol. Rather, CHIME was selected by the research team as a conceptual framework to guide the selection, evaluation, and interpretation of recovery-related outcomes. This selection was informed by the conceptual congruence between several central characteristics of the existing support model and the recovery processes represented within CHIME, including individualized planning based on participants’ life histories, identified needs, preferences, and personal goals, the promotion of autonomy, and support for participation in community life (4).

#### 2.2.1 Type of support

Support characteristics were recorded by the collaborating organization as part of routine service documentation. For each participant, a primary support and, where applicable, a secondary support was identified, and the corresponding support program, intensity, and modality were recorded.

Support intensity reflected the overall level, continuity, and extent of support required to promote personal autonomy and participation in everyday activities. Four ordered categories used in the collaborating organization’s routine service classification were retained in the study: intermittent, limited, extensive, and pervasive. This classification uses terminology closely aligned with the support-need terminology established in Spanish dependency legislation. Under Law 39/2006 and Royal Decree 174/2011, Grade I includes intermittent or limited support needs for personal autonomy, Grade II includes extensive support needs, and Grade III includes generalized support needs (20, 21). In the service classification used by the collaborating organization, intermittent and limited support were recorded as separate categories. Intermittent support referred to occasional assistance required in specific activities or situations, with a high degree of autonomy maintained in other circumstances. Limited support referred to regular support required for particular activities or during defined periods, without a need for continuous assistance. Extensive support referred to frequent and sustained assistance across several areas of everyday functioning, although not requiring continuous support throughout the day. Pervasive support represented the highest level of support intensity and referred to high intensity, continuous support required across most activities and contexts.

Support modality described the nature of the personal assistance provided and followed the four types of personal support defined in the same assessment (21). Supervision involved verbal or gestural prompting while the person performed the activity independently, as well as guidance in decision making. Partial physical assistance involved physical collaboration with the person in the partial or complete performance of an activity and could include preparation of the elements required for the person to perform it. Maximum substitution referred to situations in which the person was unable to perform the complete activity independently. Special support referred to situations in which the provision of one of the preceding forms of support was substantially hindered by exceptional health conditions and therefore required additional or specialized assistance (21).

### 2.3 Data collection procedures

The collaborating organization chose individuals that met the inclusion criteria and invited them to participate in the study. Before the baseline assessment, the research team again explained the study objectives, procedures, voluntary nature of participation, and participants’ right to withdraw, and provided an opportunity to ask questions before data collection began. Baseline assessments were conducted during April and May 2024, before the start of the predefined evaluation period associated with implementation of the updated regional support framework. The evaluation period ran from June through August 2024, and follow-up assessments were conducted during September and October 2024. Thus, all participants had completed at least the three-month evaluation period before follow-up. Given the sample size and the time required to complete the assessment battery, baseline and follow-up assessments were conducted within predefined assessment windows rather than at an identical participant-specific interval. To maintain the greatest possible temporal comparability between assessments, participants were scheduled for follow-up following the same order in which baseline assessments had been completed.

All outcome assessments were conducted individually in a private setting to preserve participants’ privacy and confidentiality. Assessments were conducted by researchers who were independent of the support team, and different researchers conducted the baseline and follow-up assessments to minimize potential bias related to the therapeutic relationship or expectations of outcome. Sociodemographic, clinical, and support-related information was obtained from the collaborating organization’s records. Sex was analyzed as recorded in these records; gender identity was not collected. Within the broader mixed-methods project, a qualitative interview was conducted before the quantitative outcome assessment at baseline. At both assessment points, the quantitative measures were administered by the researchers in a fixed order: COPM, RAS-24, and CCRQ-e, as described below.

### 2.4 Ethical issues

The project was approved by the Ethics Committee of the first author’s institution (code: 14/2023) prior to the start of the study. The study was conducted in accordance with the principles of the Declaration of Helsinki and applicable Spanish regulations. All participants were provided with detailed information about the study objectives, procedures, potential benefits, and associated risks to support an informed decision regarding participation. Written informed consent was obtained by the collaborating organization from all participants prior to their participation in the study and, where applicable, from the participant’s legal representative in accordance with the relevant procedures. This project was funded through a competitive public call.

### 2.5 Measures

The Recovery Assessment Scale (RAS-24) (22, 23) was used to evaluate the recovery process. It comprises 24 items rated on a 5-point Likert scale and includes five dimensions: Personal Confidence and Hope, Willingness to Ask for Help, Goal and Success Orientation, Reliance on Others, and No Domination by Symptoms. The total score ranges from 24 to 120, with higher scores indicating greater personal recovery. Subscale scores were calculated as the mean of the corresponding items. The Spanish version has demonstrated good psychometric properties in a clinical population with severe mental illness (22). The RAS-24 captures components across all five CHIME domains, with particularly strong representation of Empowerment, supporting its use as a measure of personal recovery within a CHIME-informed framework (16).

The perception of person-centered care was measured using the Client-Centred Rehabilitation Questionnaire (CCRQ-e) (24, 25), which assesses the extent to which rehabilitation care is perceived as responsive to users’ needs, preferences, and values. It comprises 29 items organized into seven dimensions: Decision-Making, Education, Outcome Evaluation, Family Involvement, Emotional Support, Physical Comfort, and Continuity/Coordination. Items are scored on a 5-point Likert scale, with higher scores indicating a less favorable perception of person-centered care. Dimension scores were calculated as the mean of applicable items, and the overall CCRQ-e score as the unweighted mean of the dimension scores. Responses coded as 0 (not applicable) or 99 (no response) were excluded from score calculation. The Family Involvement dimension was optional and was completed only when applicable to the participant’s situation. At the follow-up assessment, this dimension was re-administered to all participants, regardless of whether it had been completed at baseline, in accordance with the instrument’s administration procedure. Within the CHIME-informed evaluation, the CCRQ-e was used as a complementary measure of participants’ perceptions of person-centered care rather than as a direct measure of any specific CHIME process.

The Canadian Occupational Performance Measure (COPM) (26, 27) was used to evaluate changes in occupational performance and satisfaction. This individualized measure enables participants to identify and prioritize up to five meaningful activities in which they experience difficulties and to rate both their performance and satisfaction for each activity on a 1–10 scale, with higher scores indicating better perceived performance or satisfaction. In accordance with standard COPM scoring procedures, for each participant and assessment point, overall performance and satisfaction scores were calculated as the arithmetic mean of the valid ratings across all personally identified priority activities. Its individualized focus on occupational performance and participation problems in activities identified as relevant by the participant was considered conceptually most closely related to Meaning, particularly meaningful activities and personal goals, and its use within recovery-oriented mental health practice has previously been described (4, 28).

Taken together, the RAS-24, CCRQ-e, and COPM provided complementary participant-reported information on personal recovery, perceived person-centered care, and individualized ratings of occupational performance and satisfaction, respectively. Their interpretation within this CHIME-informed evaluation was based on conceptual correspondence rather than on the assumption that any instrument directly or exclusively measures a particular CHIME process or CHIME as a whole.

### 2.6 Data analysis

Statistical analyses were performed using IBM SPSS Statistics, version 31.0 (IBM Corp., Armonk, NY, USA). Continuous variables were summarized using means and standard deviations, and categorical variables using frequencies and percentages. Sociodemographic and clinical characteristics were also examined by sex. Age was compared between male and female participants using an independent-samples *t*-test, with Cohen’s *d* as the effect-size measure, whereas associations between sex and categorical clinical or support characteristics were examined using Pearson’s chi-square tests and Cramer’s *V*.

Diagnostic information was based on psychiatrist-established diagnoses according to the Diagnostic and Statistical Manual of Mental Disorders, Fifth Edition, Text Revision (DSM-5-TR) (29), as provided by the collaborating organization. For inferential analyses, primary diagnoses were grouped into three mutually exclusive categories: schizophrenia spectrum and other psychotic disorders, depressive and bipolar disorders, and other psychiatric disorders. Depressive and bipolar disorders were combined within a broader mood-disorder grouping both because the individual diagnostic categories were too small to support stable estimates in separate adjusted analyses and because there is an established nosological basis for considering them within a broader mood-disorder category. Depressive and bipolar disorders were historically included within the same Mood Disorders category in the Diagnostic and Statistical Manual of Mental Disorders, Fourth Edition (DSM-IV) (30), whereas DSM-5-TR places depressive and bipolar disorders in distinct diagnostic chapters (29). The International Classification of Diseases, 11th Revision (ICD-11) likewise retains Mood Disorders as a higher-level grouping that includes depressive and bipolar disorders (31). Nevertheless, these conditions are clinically distinct; the combined grouping was therefore used for analytical purposes, while specific primary and co-occurring DSM-5-TR diagnoses were retained for descriptive analyses.

The original primary support programs were also presented descriptively but were grouped for adjusted analyses into two broader domains: vocational and economic inclusion supports (training and employment guidance, pre-employment workshops, and self-employment) and community living and social inclusion supports (supervised housing, housing for young adults leaving care, social reintegration for people with criminal records, and leisure and free-time support). This grouping was informed by World Health Organization guidance on community mental health services, which emphasizes community inclusion and the importance of linking community-based mental health support with key areas of everyday life, including housing, education, employment, and social protection (8).

The 7-dimension CCRQ-e total, including Family Involvement, was used as the primary CCRQ-e outcome. Because Family Involvement was optional and had lower data availability, a 6-dimension CCRQ-e total excluding this dimension was additionally examined as a sensitivity outcome. Unadjusted pre–post changes in the four primary outcomes (RAS-24 total score, CCRQ-e 7-dimension total score, COPM performance mean score, and COPM satisfaction mean score) were initially examined using paired-samples *t*-tests. Mean changes were calculated as follow-up minus baseline, and 95% confidence intervals were reported. As a complementary descriptive analysis of individual COPM change, the number and proportion of participants with paired data showing an increase of at least 2 points in performance and satisfaction were calculated, using the historically cited 2-point COPM benchmark for contextual interpretation (32). Effect sizes were expressed as Cohen’s *d*, calculated as the mean paired difference divided by the standard deviation of the paired differences. A paired-samples *t*-test was also conducted for the 6-dimension CCRQ-e sensitivity outcome. Secondary paired analyses were conducted for the five RAS-24 subscales and seven CCRQ-e dimensions; Holm’s procedure was applied separately within each family of tests.

The primary longitudinal analyses were performed using separate linear mixed models for RAS-24 total score, CCRQ-e 7-dimension total score, COPM performance mean score, and COPM satisfaction mean score. Time (baseline vs. follow-up) was specified as a repeated measure within participant, with an unstructured covariance matrix. Fixed effects included time, sex, age centered at the sample mean, primary diagnostic group, primary support program domain, primary support intensity, secondary support receipt, and the time × sex interaction. Primary support modality was not entered simultaneously with support intensity in the main models; instead, sensitivity models replaced primary support intensity with primary support modality while retaining all other model terms. Models were estimated using restricted maximum likelihood (REML), and denominator degrees of freedom were estimated using the Satterthwaite approximation. Adjusted baseline and follow-up estimated marginal means, adjusted pre–post changes with 95% confidence intervals, and Type III tests of fixed effects were obtained. Holm’s procedure was applied across the four primary tests of the time effect.

Sex-specific estimated marginal means were obtained from the primary models to characterize adjusted pre–post trajectories for female and male participants. Exploratory models further examined whether longitudinal changes differed jointly according to sex and primary diagnostic group by adding time × diagnostic group, sex × diagnostic group, and time × sex × diagnostic group interactions to the primary model specification. These interaction analyses were considered exploratory.

For non-time fixed effects showing a significant global Type III test in the primary mixed models, estimated marginal means were obtained to characterize the direction of the association. Pairwise comparisons were performed where appropriate and adjusted using the Bonferroni procedure. Omnibus *p*-values for non-time fixed effects were not adjusted for multiplicity across effects or outcomes. These estimated marginal means were averaged across assessment times and were therefore interpreted as adjusted between-group differences rather than differential changes over time.

Associations between primary diagnostic group and primary support modality and intensity were examined using Fisher–Freeman–Halton exact tests, with Cramer’s *V* reported as the effect-size measure.

Several sensitivity analyses were undertaken to assess the robustness of the primary findings. First, the primary mixed models were re-estimated replacing support intensity with support modality. Second, baseline-adjusted analysis of covariance (ANCOVA) models were fitted for each follow-up outcome, adjusting for the corresponding baseline score, sex, age, primary diagnostic group, primary support program domain, secondary support receipt, and either primary support intensity or primary support modality. Homogeneity of error variances in the ANCOVA models was assessed using Levene’s test. Third, the CCRQ-e mixed model was repeated using the 6-dimension score excluding Family Involvement. Finally, Wilcoxon signed-rank tests were conducted as nonparametric sensitivity analyses for the four primary pre–post outcomes. Holm’s procedure was applied across these four Wilcoxon tests, and effect size was calculated as r=∣Z∣/√n , where n represents the number of complete pairs.

### 2.7 Missing-data handling

No missing values were imputed. Missing data were handled according to the requirements of each analysis. Paired-samples *t*-tests, Wilcoxon signed-rank tests, and baseline-adjusted ANCOVA analyses were based on complete cases for the specific outcome being analyzed. Participants with data at only one assessment point were excluded from the corresponding paired or ANCOVA analysis but remained in the overall sample and in any other analyses for which valid data were available.

In contrast, the linear mixed models used all available outcome observations and therefore retained participants with valid data at only one assessment point; a complete pre–post pair was not required for inclusion. This accounts for differences between the numbers of complete pairs reported in the unadjusted analyses and the numbers of participants and observations included in the mixed models.

For the RAS-24 total score, 158 complete pre–post pairs were available, whereas the mixed model used 317 observations from all 159 participants. For the 7-dimension CCRQ-e total, data availability was lower because the Family Involvement dimension was optional and was completed only when applicable. Consequently, the paired analysis included 100 complete pre–post pairs, whereas the mixed model used 246 available observations from 146 participants. A 6-dimension CCRQ-e score excluding Family Involvement was therefore examined as a sensitivity analysis to assess whether this source of reduced data availability affected the findings; this sensitivity model included 316 observations from 159 participants.

For the COPM, participants who did not identify any priority activity did not generate a valid mean score. One participant did not identify a priority activity at either assessment, and one additional participant had a valid baseline score but did not complete the follow-up assessment. Accordingly, paired COPM analyses included 157 complete pairs, whereas the mixed models retained all available COPM observations (158 participants; 315 observations). At each assessment, participants with at least one valid identified priority contributed a participant-level mean calculated from all valid priority ratings.

### 2.8 Model diagnostics

Model diagnostics for the primary linear mixed models were assessed by visual inspection of residual histograms, normal Q–Q plots, and residual-versus-fitted plots. No marked systematic patterns or severe heteroscedasticity were observed. Mild departures from normality were noted in the distribution tails, particularly for the CCRQ-e model. A small number of extreme residuals corresponded to valid observed scores and were therefore retained in the analyses.

All statistical tests were two-tailed, with a significance level of *p* < 0.05. Where multiplicity adjustments were applied, both unadjusted and adjusted *p*-values are reported as specified above.

## 3 Results

A total of 159 participants were included in the study, most of whom were male (67.9%; *n* = 108), with a mean age of 49.07 years (SD = 12.38). Age did not differ significantly by sex. Schizophrenia spectrum and other psychotic disorders constituted the largest primary diagnostic group (40.9%), followed by other psychiatric disorders (35.8%) and depressive and bipolar disorders (23.3%). The distribution of primary diagnostic groups differed significantly by sex (*χ*^2^(2) = 7.087, *p* = 0.029, Cramer’s *V* = 0.211): schizophrenia spectrum and other psychotic disorders were more frequent among male participants (46.3% vs. 29.4%), whereas depressive and bipolar disorders were more frequent among female participants (35.3% vs. 17.6%; Table 1). Schizophrenia was the most frequent individual primary diagnosis (*n* = 38, 23.9%; Supplementary Table S1, Panel A); primary and co-occurring diagnoses are additionally presented in Supplementary Table S1, Panel B.

**Table 1:** Sociodemographic and clinical characteristics of the sample overall and by sex.

Variable	Total *N* = 159	Male *n* = 108	Female *n* = 51	Statistic	*p*	Effect size
Age, years, mean (SD)	49.07 (12.38)	48.91 (12.11)	49.41 (13.06)	*t* = 0.239	0.811	Cohen’s *d* = 0.04
Primary diagnostic group, *n* (%)				*χ*^2^(2) = 7.087	0.029	Cramer’s *V* = 0.211
Schizophrenia spectrum and other psychotic disorders	65 (40.9)	50 (46.3)	15 (29.4)			
Depressive and bipolar disorders	37 (23.3)	19 (17.6)	18 (35.3)			
Other psychiatric disorders	57 (35.8)	39 (36.1)	18 (35.3)			

Values are presented as *n* (%) unless otherwise indicated. Percentages were calculated within each sex category. Differences by sex were assessed using the independent-samples *t*-test for age and Pearson’s chi-square test for the association between sex and primary diagnostic group. Effect sizes are reported as Cohen’s *d* and Cramer’s *V*, respectively. *n*, number of participants; *N*, total sample size; SD, standard deviation; *t*, independent-samples *t* statistic; *χ*^2^, Pearson’s chi-square statistic; *p*, probability value; *d*, Cohen’s effect size; *V*, Cramer’s *V* effect size.

The most common primary support programs were social reintegration for people with criminal records (54.1%), pre-employment workshops (30.2%), and training and employment guidance (11.3%). Overall, 57.2% of participants received community living and social inclusion supports and 42.8% received vocational and economic inclusion supports. Extensive support was the most frequent intensity level (35.2%), and maximum substitution was the most frequent support modality (32.7%). Secondary support was received by 73 participants (45.9%), most commonly leisure and free-time activities, personal assistance, and training and employment guidance (Table 2; Supplementary Table S2). No significant sex differences were observed in the grouped support characteristics (all *p* ≥ 0.150; Table 2).

**Table 2:** Community support characteristics overall and by sex.

Support characteristic	Total *N* = 159	Male *n* = 108	Female *n* = 51	Statistic	*p*	Effect size
Primary support program, *n* (%)				–	–	–
Training and employment guidance	18 (11.3)	14 (13.0)	4 (7.8)			
Pre-employment workshops	48 (30.2)	28 (25.9)	20 (39.2)			
Self-employment	2 (1.3)	0	2 (3.9)			
Supervised housing	3 (1.9)	3 (2.8)	0			
Housing for young adults leaving care	1 (0.6)	1 (0.9)	0			
Social reintegration for people with criminal records	86 (54.1)	61 (56.5)	25 (49.0)			
Leisure and free time	1 (0.6)	1 (0.9)	0			
Primary support program domain, *n* (%)				*χ*^2^(1) = 2.069	0.150	Cramer’s *V* = 0.114
Vocational and economic inclusion supports	68 (42.8)	42 (38.9)	26 (51.0)			
Community living and social inclusion supports	91 (57.2)	66 (61.1)	25 (49.0)			
Primary support intensity, *n* (%)				*χ*^2^(3) = 3.415	0.332	Cramer’s *V* = 0.147
Intermittent	44 (27.7)	29 (26.9)	15 (29.4)			
Limited	43 (27.0)	26 (24.1)	17 (33.3)			
Extensive	56 (35.2)	43 (39.8)	13 (25.5)			
Pervasive	16 (10.1)	10 (9.3)	6 (11.8)			
Primary support modality, *n* (%)				*χ*^2^(3) = 2.281	0.516	Cramer’s *V* = 0.120
Supervision	47 (29.6)	32 (29.6)	15 (29.4)			
Partial physical assistance	44 (27.7)	27 (25.0)	17 (33.3)			
Maximum substitution	52 (32.7)	39 (36.1)	13 (25.5)			
Special support	16 (10.1)	10 (9.3)	6 (11.8)			
Secondary support received, *n* (%)				*χ*^2^(1) = 0.233	0.629	Cramer’s *V* = 0.038
No	86 (54.1)	57 (52.8)	29 (56.9)			
Yes	73 (45.9)	51 (47.2)	22 (43.1)			

Values are presented as *n* (%). Percentages were calculated within each sex category. Pearson’s chi-square tests were used to examine associations between sex and grouped support characteristics. Effect sizes are reported as Cramer’s *V*. Original primary support program categories are presented descriptively; the grouped primary support program domain was used for inferential analyses. *n*, number of participants; *N*, total sample size; *χ*^2^, Pearson’s chi-square statistic; *p*, probability value; *V*, Cramer’s *V* effect size.

Primary diagnostic group was significantly associated with both primary support modality (Fisher–Freeman–Halton exact *p* = 0.004, Cramer’s *V* = 0.242) and support intensity (*p* = 0.005, Cramer’s *V* = 0.242). Participants with schizophrenia spectrum and other psychotic disorders more frequently received maximum-substitution support (44.6%) and extensive support (47.7%), whereas participants with depressive and bipolar disorders more frequently received supervision (40.5%) or partial physical assistance (37.8%) and limited (40.5%) or intermittent (35.1%) support (Supplementary Table S3).

In the unadjusted paired analyses, no significant pre–post change was observed in RAS-24 total scores (mean change = 0.54, 95% CI −1.95 to 3.03, *p* = 0.670, Cohen’s *d_z_* = 0.03) or in the 7-dimension CCRQ-e total score (mean change = 0.09, 95% CI −0.06 to 0.25, *p* = 0.231, *d_z_* = 0.12). The 6-dimension CCRQ-e score excluding Family Involvement yielded a similarly non-significant result (*p* = 0.238). In contrast, COPM scores increased significantly for both performance (mean change = 0.75, 95% CI 0.50–0.99, *p* < 0.001, *d_z_* = 0.48) and satisfaction (mean change = 0.85, 95% CI 0.60–1.11, *p* < 0.001, *d_z_* = 0.53; Table 3). At the individual level, 29 of 157 participants with paired COPM data (18.5%) showed an increase of at least 2 points in performance, and 30 of 157 (19.1%) showed an increase of at least 2 points in satisfaction. At baseline, one participant did not identify any COPM priority activity; 25 participants identified one priority, 51 identified two, 44 identified three, 17 identified four, and 21 identified all five priorities.

**Table 3:** Unadjusted pre–post changes in recovery, person-centered care, and occupational performance.

Outcome	Complete pairs *n*	Baseline mean (SD)	Follow-up mean (SD)	Mean change post–pre (95% CI)	*t*	*p*	Cohen’s *d_z_*
RAS-24 total score	158	89.46 (16.74)	90.00 (17.82)	0.54 (−1.95 to 3.03)	0.427	0.670	0.03
CCRQ-e total, 7 dimensions	100	1.78 (0.66)	1.88 (0.76)	0.09 (−0.06 to 0.25)	1.204	0.231	0.12
CCRQ-e total, 6 dimensions excluding Family Involvement (sensitivity analysis)	157	1.78 (0.69)	1.85 (0.74)	0.07 (−0.05 to 0.19)	1.185	0.238	0.09
COPM performance mean score	157	3.21 (1.66)	3.96 (2.07)	0.75 (0.50–0.99)	5.959	<0.001	0.48
COPM satisfaction mean score	157	2.95 (1.48)	3.80 (1.99)	0.85 (0.60–1.11)	6.598	<0.001	0.53

Values are presented as mean (SD). Mean changes were calculated as follow-up minus baseline; therefore, positive values indicate higher scores at follow-up. Paired-samples *t*-tests were used for pre–post comparisons. Cohen’s *d_z_* was calculated as the mean paired change divided by the standard deviation of the paired changes. For the CCRQ-e, higher scores indicate a less favorable perception of person-centered care. The 7-dimension CCRQ-e total was the primary CCRQ-e outcome. A 6-dimension total excluding Family Involvement was examined as a sensitivity analysis because the optional Family Involvement dimension reduced the number of complete observations available for the 7-dimension total. RAS-24, Recovery Assessment Scale-24; CCRQ-e, Spanish version of the Client-Centred Rehabilitation Questionnaire; COPM, Canadian Occupational Performance Measure; *n*, number of complete pairs; SD, standard deviation; CI, confidence interval; *t*, paired-samples *t* statistic; *p*, probability value; *d_z_*, Cohen’s effect size for paired observations.

The primary linear mixed models confirmed these findings after adjustment for sex, age, primary diagnostic group, primary support program domain, primary support intensity, secondary support receipt, and the time × sex interaction. Significant increases over time were observed for COPM performance (adjusted change = 0.73, 95% CI 0.46–0.99, Holm-adjusted *p* < 0.001) and COPM satisfaction (adjusted change = 0.86, 95% CI 0.59–1.13, Holm-adjusted *p* < 0.001). No significant time effects were found for RAS-24 (adjusted change = 0.85, 95% CI −1.81 to 3.52, *p* = 0.529) or the 7-dimension CCRQ-e total score (adjusted change = 0.11, 95% CI −0.05 to 0.26, Holm-adjusted *p* = 0.361; Table 4).

**Table 4:** Adjusted longitudinal changes in recovery, person-centered care, and occupational performance.

Outcome	Participants/observations	Adjusted baseline mean (95% CI)	Adjusted follow-up mean (95% CI)	Adjusted change post–pre (95% CI)	*p*, time	Holm-adjusted *p*, time	*p*, time × sex
RAS-24 total score	159/317	88.42 (85.25–91.58)	89.27 (85.99–92.54)	0.85 (−1.81 to 3.52)	0.529	0.529	0.490
CCRQ-e total, 7 dimensions	146/246	1.82 (1.66–1.97)	1.92 (1.77–2.08)	0.11 (−0.05 to 0.26)	0.181	0.361	0.753
COPM performance mean score	158/315	3.01 (2.70–3.32)	3.74 (3.36–4.11)	0.73 (0.46–0.99)	<0.001	<0.001	0.716
COPM satisfaction mean score	158/315	2.74 (2.47–3.01)	3.60 (3.24–3.96)	0.86 (0.59–1.13)	<0.001	<0.001	0.900

Adjusted means are estimated marginal means from separate linear mixed models adjusted for time, sex, age, primary diagnostic group, primary support program domain, primary support intensity, secondary support receipt, and the time × sex interaction. Changes were calculated as follow-up minus baseline; positive values indicate higher follow-up scores, whereas higher CCRQ-e scores indicate less favorable person-centered care. Holm’s procedure was applied across the four primary tests of the time effect. All available observations were used; therefore, sample sizes vary across outcomes. RAS-24, Recovery Assessment Scale-24; CCRQ-e, Spanish version of the Client-Centred Rehabilitation Questionnaire; COPM, Canadian Occupational Performance Measure; CI, confidence interval; *p*, probability value.

No evidence of differential change over time by sex was found for any of the four primary outcomes (time × sex: RAS-24, *p* = 0.490; CCRQ-e, *p* = 0.753; COPM performance, *p* = 0.716; COPM satisfaction, *p* = 0.900; Table 5, Panel A). Exploratory three-way time × sex × diagnostic group interactions were also non-significant for RAS-24, CCRQ-e, and COPM performance; the interaction for COPM satisfaction approached but did not reach statistical significance (*p* = 0.055; Table 5, Panel B).

**Table 5:** Sex-specific adjusted means from primary linear mixed models and exploratory sex-by-diagnosis interaction tests.

Panel A. Sex-specific adjusted means from the primary linear mixed models					
Outcome	Female baseline mean (95% CI)	Female follow-up mean (95% CI)	Male baseline mean (95% CI)	Male follow-up mean (95% CI)	*p*, time × sex
RAS-24 total score	87.06 (82.27–91.86)	88.85 (83.86–93.83)	89.77 (86.00–93.54)	89.69 (85.79–93.58)	0.490
CCRQ-e total, 7 dimensions	1.80 (1.57–2.04)	1.93 (1.69–2.17)	1.83 (1.65–2.01)	1.91 (1.73–2.09)	0.753
COPM performance mean score	3.01 (2.55–3.48)	3.69 (3.11–4.27)	3.01 (2.63–3.38)	3.78 (3.34–4.23)	0.716
COPM satisfaction mean score	2.72 (2.31–3.12)	3.59 (3.04–4.15)	2.76 (2.43–3.10)	3.61 (3.18–4.03)	0.900

Panel B. Exploratory time × sex × primary diagnostic group interaction tests					
Outcome	Interaction tested	*F*	Numerator *df*	Denominator *df*	*p*
RAS-24 total score	Time × sex × diagnostic group	0.045	2	152.358	0.956
CCRQ-e total, 7 dimensions	Time × sex × diagnostic group	2.241	2	110.436	0.111
COPM performance mean score	Time × sex × diagnostic group	1.444	2	151.076	0.239
COPM satisfaction mean score	Time × sex × diagnostic group	2.951	2	151.057	0.055

Panel A presents estimated marginal means from the primary linear mixed models adjusted for time, sex, age, primary diagnostic group, primary support program domain, primary support intensity, secondary support receipt, and the time × sex interaction. Panel B presents the time × sex × primary diagnostic group interaction from exploratory models that additionally included the corresponding time × diagnostic group and sex × diagnostic group interactions. Panel B *p*-values are unadjusted and should be interpreted as exploratory. Models used all available observations. Higher CCRQ-e scores indicate less favorable person-centered care. RAS-24, Recovery Assessment Scale-24; CCRQ-e, Spanish version of the Client-Centred Rehabilitation Questionnaire; COPM, Canadian Occupational Performance Measure; CI, confidence interval; *F*, *F* statistic; *df*, degrees of freedom; *p*, probability value.

Among the non-time fixed effects in the primary models, several adjusted between-group differences were observed (Supplementary Table S7). RAS-24 scores averaged across assessment times were higher among participants receiving vocational and economic inclusion supports than among those receiving community living and social inclusion supports (adjusted means 91.49 vs. 86.19; adjusted mean difference = 5.31, 95% CI 0.24–10.37, *p* = 0.040). Primary diagnostic group was associated with COPM performance (omnibus *p* = 0.020): participants with schizophrenia spectrum and other psychotic disorders had higher adjusted mean performance scores than those with depressive and bipolar disorders (3.91 vs. 2.95; adjusted mean difference = 0.95, 95% CI 0.07–1.84, Bonferroni-adjusted *p* = 0.030), while the remaining pairwise comparisons were non-significant. Primary diagnostic group was also associated with COPM satisfaction (omnibus *p* = 0.003): the schizophrenia spectrum and other psychotic disorders group had higher adjusted satisfaction scores than both the depressive and bipolar group (3.75 vs. 2.76; adjusted mean difference = 0.99, 95% CI 0.21–1.77, *p* = 0.007) and the other psychiatric disorders group (3.75 vs. 3.01; adjusted mean difference = 0.74, 95% CI 0.08–1.40, *p* = 0.023). In addition, participants not receiving secondary support had higher adjusted COPM satisfaction scores than those receiving secondary support (3.47 vs. 2.87; adjusted mean difference = 0.61, 95% CI 0.06–1.16, *p* = 0.031). These estimated marginal means represent differences averaged across assessment times and should therefore not be interpreted as differential pre–post changes.

Secondary analyses did not identify statistically significant changes in any RAS-24 subscale or CCRQ-e dimension after Holm correction (Supplementary Table S4). Education showed an unadjusted increase in CCRQ-e scores (*p* = 0.025), but this did not remain significant after correction for multiple comparisons (Holm-adjusted *p* = 0.178). The sensitivity analysis excluding the optional Family Involvement dimension likewise showed no significant CCRQ-e time effect (*p* = 0.156) or time × sex interaction (*p* = 0.379; Supplementary Table S8). Nonparametric sensitivity analyses yielded the same substantive conclusions: no significant change was observed in RAS-24 (*p* = 0.883; Holm-adjusted *p* = 0.883) or CCRQ-e (*p* = 0.163; Holm-adjusted *p* = 0.326), whereas increases in COPM performance and satisfaction remained statistically significant (both Holm-adjusted *p* < 0.001; Supplementary Table S9). Results were also materially unchanged when primary support modality replaced support intensity in the mixed models (Supplementary Table S5), and baseline-adjusted ANCOVA sensitivity models yielded similar follow-up estimates when adjusted for either primary support intensity or primary support modality (Supplementary Table S6). Levene’s tests did not indicate violations of the homogeneity of error variances assumption in any of the ANCOVA sensitivity models (all *p* ≥ 0.142; Supplementary Table S6).

## 4 Discussion

The aim of this study was to examine, within a CHIME-informed conceptual framework, changes in personal recovery, perceived person-centered care, occupational performance, and satisfaction with performance among adults receiving routine community mental health support. The study provides evidence from real-world service delivery in a heterogeneous sample of 159 adults supported within the public social services system. Its main contribution lies in examining complementary recovery-related outcomes that reflect personal recovery, occupational performance and satisfaction in activities prioritized because participants experienced problems in occupational performance or participation, and the perceived experience of person-centered care. No uniform pattern of improvement was observed across these outcomes. Statistically significant increases were detected in COPM performance and satisfaction, although the adjusted mean changes were less than one point on the 10-point scales, whereas no statistically significant longitudinal changes were detected in personal recovery or perceived person-centered care during the evaluation period. These findings indicate that different components relevant to recovery can follow different trajectories within the same period of community support and highlight the value of evaluating services using outcomes that capture both person-defined priorities and broader experiences of recovery and care.

The differentiated pattern observed is consistent with the distinction, outlined in the Background, between related but non-interchangeable dimensions of recovery. Winsper et al. showed that recovery-oriented interventions may be associated with functional, existential, and social recovery outcomes through partly distinct mechanisms (5), while Van Eck et al. found only small-to-moderate associations between personal recovery and components of clinical recovery in people with schizophrenia spectrum disorders (6). Thus, the coexistence of increases in occupational performance and satisfaction with stable RAS-24 and CCRQ-e scores is compatible with a differentiated pattern across complementary recovery-related outcomes.

The selection of outcome measures should be understood from this complementary perspective. CHIME was used as a conceptual framework to guide outcome selection, evaluation, and interpretation, rather than as an intervention protocol or psychometric instrument. Vogel et al. mapped RAS items across all five CHIME domains, although the distribution was uneven and Empowerment was particularly represented (16). Penas et al. likewise noted that measures including the RAS do not map adequately onto the complete five-process CHIME framework (17). These findings support avoiding a one-to-one correspondence between any single scale and CHIME as a whole. This interpretation is also consistent with the systematic review by Felix et al., which identified 25 self-report measures of personal recovery and found that no measure had sufficiently strong evidence across all evaluated measurement properties (18).

Two non-exclusive explanations should be considered for this differentiated pattern. First, the constructs may genuinely follow different short-term trajectories: changes in performance and satisfaction in participant-selected activities may occur without concurrent change in broader personal recovery or perceived person-centered care. Second, measurement responsiveness may have contributed. The COPM is individualized and directly anchored to activities that each participant identified as problematic and important, whereas the RAS-24 and CCRQ-e use fixed standardized items to assess broader constructs. Consequently, the COPM may be more closely aligned with short-term changes in personally prioritized activities. The present study cannot distinguish the relative contribution of genuine construct-specific change from differential measurement responsiveness, and both explanations should be considered when interpreting the findings.

The CHIME-organized sections that follow provide a theoretically informed interpretation of complementary outcomes and should not be understood as separate empirical or quantitative assessments of the five CHIME processes.

### 4.1 Meaning

Among the CHIME processes, Meaning provides the clearest conceptual lens for interpreting the main change observed in this study. Within CHIME, Meaning in life encompasses aspects such as purpose, valued roles, meaningful activities, and personal goals (4). The COPM was selected to provide an individualized assessment in which participants identify and prioritize problems in occupational performance and participation in activities relevant to them and subsequently rate their performance and satisfaction with performance in the prioritized activities (26, 27). This feature is particularly relevant within a recovery framework because the assessment is anchored in areas of everyday life identified as important by the participant rather than in a uniform set of externally predetermined tasks.

The use of the COPM in this context also has specific support in mental health. Kirsh and Cockburn examined its application in psychiatric rehabilitation and recovery-oriented practice and highlighted its role in supporting goal setting, participation, resumption of life roles, and inclusion in environments of the person’s choice (28). More broadly, Winsper et al. identified movement toward personally meaningful goals as a central feature of the recovery paradigm (5). The observed increases in occupational performance and satisfaction are therefore conceptually informative because they concern activities prioritized from problems in occupational performance or participation identified by participants themselves, rather than a standardized set of activities selected by professionals or researchers.

However, the magnitude of the COPM changes requires cautious interpretation. The adjusted mean increases were 0.73 points for performance and 0.86 points for satisfaction on the 10-point COPM scales. For context, these group-level mean changes were below the 2-point change historically cited for the COPM as indicating clinically significant change. However, a review specifically examining the empirical basis of this criterion concluded that the 2-point benchmark should not be regarded as a universally applicable threshold for clinical significance (32). At the individual level, 29 of 157 participants with paired COPM data (18.5%) showed an increase of at least 2 points in performance, and 30 of 157 (19.1%) showed an increase of at least 2 points in satisfaction. More recent evidence further supports a context-dependent interpretation, with a systematic review reporting estimates of minimal important change ranging from 0.20 to 3.20 points for performance and from 1.45 to 3.20 points for satisfaction across different populations and study conditions (33). Taken together, these findings indicate statistically detectable group-level increases in occupational performance and satisfaction alongside substantial variability in the magnitude of individual change. The proportions exceeding the historically cited 2-point benchmark provide useful descriptive information but should not be interpreted as definitive estimates of the proportion of participants experiencing clinically important improvement.

This interpretation also has relevance for community mental health service evaluation. In their systematic review of community-based models for people with persistent and complex mental health needs, Harvey et al. found that emerging models supporting personal and functional recovery were characterized by placing consumer goals and priorities at the center of care. They also emphasized the need for models of care to evolve and adapt to heterogeneous needs (14). The COPM findings can therefore be considered not only at the level of an individual outcome measure but also within the broader question of which outcomes are informative when assessing person-centered community support.

In relation to the process through which priorities were identified, it is noteworthy that 77 participants, representing 48.4% of the sample, identified fewer than three priorities at baseline. This does not indicate incomplete COPM administration, nor does it imply that these participants had no other relevant activities or participation concerns. It does, however, suggest that identifying, formulating, and prioritizing problems in occupational performance and participation may itself be a complex process for a substantial proportion of people receiving mental health support.

The systematic integrative review by Stewart et al., which included 54 studies across mental health settings, showed that goal planning is influenced by personal, interpersonal, and organizational factors. Collaboration and concordance between service users and professionals were prominent themes, and individualized, recovery-oriented, and collaborative goal planning was recommended, although it was not consistently implemented in practice (34). Identification of priorities should therefore not be viewed simply as asking a person what they want to achieve; for some individuals, the process may require exploration, reflection, dialog, and professional facilitation.

Processes related to self-reflection and metacognition may also be relevant for some participants. In people with schizophrenia, metacognition has been associated with psychosocial outcomes including motivation, self-evaluation, and vocational functioning (35). The capacity to integrate information about oneself and one’s experiences may influence how some individuals recognize difficulties and formulate future goals. Because schizophrenia spectrum and other psychotic disorders accounted for an important, but not exclusive, proportion of the sample, this literature provides one possible explanation for some participants and should not be generalized to everyone who identified a small number of priorities.

Equally, identifying few priorities should not be interpreted solely in terms of individual difficulties. Moxham et al. showed that people living with serious mental illness were able to establish self-defined goals and subsequently assess their attainment when goal setting was explicitly facilitated within a structured context (36). Stewart et al. similarly emphasize collaboration and the professional skills required to support effective goal planning (34). The number of priorities identified in the present study therefore highlights the importance of the process through which interests, roles, occupational performance and participation problems, aspirations, and life projects are explored, particularly when priorities are not readily articulated.

This issue is especially relevant for support models that take the person’s life project as their starting point. Person-centered care involves more than asking for goals; it also requires conditions that enable people to explore which activities, roles, and areas of life matter to them, recognize barriers to performance and participation, and progressively translate these priorities into goals that can be supported and reviewed (34). From this perspective, the process through which goals emerge is itself part of the individualization of support.

### 4.2 Connectedness

Connectedness encompasses relationships, support from others, a sense of belonging, and participation in the community as core processes of recovery (4). This dimension is particularly relevant to the program evaluated because a substantial proportion of support was provided in areas related to community living and social inclusion, social reintegration, employment, leisure, and personal assistance. Social determinants, opportunities for participation, and social networks are important to the health and wellbeing of people with mental disorders (37), while community integration, social support, and independence have been associated with personal recovery among people living in the community (38).

The stability of the relational dimensions of the CCRQ-e provides a complementary perspective. The availability of community-based supports aimed at inclusion and participation is not equivalent to the subjective experience of connectedness, emotional support, family involvement, or continuity of care. Connectedness within CHIME concerns the lived relational and belonging dimensions of recovery rather than simply the physical availability of community services (4). The findings therefore underline the value of considering both the context in which support is provided and how people experience their relationships, belonging, and participation within that context.

### 4.3 Hope and optimism about the future

No statistically significant longitudinal change was detected in overall personal recovery or in the RAS-24 dimensions most closely related to confidence, hope, and goal orientation. Hope within CHIME encompasses belief in the possibility of recovery, positive expectations for the future, motivation to change, and the ability to envisage future goals and possibilities (1, 4). It is therefore broader than improved performance in a particular activity.

The literature indicates that personal recovery does not necessarily change in parallel with other outcomes. Van Eck et al. found significant but only partial relationships between personal and clinical recovery in schizophrenia spectrum disorders (6). Similarly, Mitsunaga-Ohmuro and Ohmuro reported clinical changes among people with psychotic disorders during hospitalization without simultaneous significant changes in several indicators of personal recovery, including the RAS (39). Although the setting and observation period differ from those of the present study, these findings illustrate that change in one aspect of a person’s experience need not be accompanied by simultaneous change in broader personal recovery.

Harvey et al. also emphasize the importance of study timeframes and recommend extended follow-up when examining recovery outcomes in community models of care (14). Accordingly, the absence of statistically detectable change in RAS-24 scores in the present study is most appropriately interpreted as an absence of detectable change in personal recovery during the observed evaluation interval. The absence of statistically detectable change should not automatically be interpreted as a beneficial outcome, but neither does it diminish the changes observed in other areas.

A plausible interpretation is that perceiving better performance in specific activities relevant to one’s life represents a different level of change from changing a broader appraisal of hope, confidence, and one’s recovery trajectory. Winsper et al. distinguish functional, social, and existential recovery outcomes and identify partly different mechanisms through which these may develop (5). The pattern observed here therefore supports assessing everyday changes alongside broader personal recovery constructs rather than assuming that either can substitute for the other.

### 4.4 Identity

Identity refers to developing or rebuilding a valued sense of self that is not defined solely by mental illness and includes the development of positive identity, valued roles, and a life beyond the diagnostic label (1, 4). Recent evidence also supports the broadly transdiagnostic relevance of these processes. Lases et al. found that all five CHIME processes, including Identity, were richly represented in recovery narratives across different diagnostic groups (3).

Identity is conceptually relevant to the support model evaluated because planning begins with the person’s life history, needs, preferences, and personal goals. The COPM was selected primarily because of its conceptual relationship with Meaning rather than as a specific measure of Identity. Nevertheless, its individualized process of identifying and prioritizing problems in occupational performance and participation in activities relevant to the person may also connect with roles, interests, responsibilities, and life projects. Kirsh and Cockburn specifically note that the COPM can support participation, resumption of life roles, and inclusion within recovery-oriented practice (28).

From this perspective, identifying relevant activities is not simply a matter of selecting tasks on which to intervene. These activities may represent areas of life through which people enact responsibilities, interests, capacities, and valued roles. Recovery involves developing a life and identity beyond illness (1, 4). The biographical basis of support planning and the use of priorities defined by participants therefore bring elements of roles and life projects into the broader interpretation of recovery, without treating COPM scores as direct evidence of change in Identity.

### 4.5 Empowerment and person-centered care

The coexistence of increased occupational performance and satisfaction with no statistically detectable longitudinal change in personal recovery and perceived person-centered care represents another conceptually relevant finding. Within CHIME, Empowerment includes personal responsibility, control over one’s life, recognition of strengths, and active participation in recovery (4, 17). The RAS-24 has substantial representation of Empowerment-related content (16), whereas the CCRQ-e captures the person’s perception of several dimensions of person-centered rehabilitation (24).

The pattern observed suggests that changes in participant-rated occupational performance and satisfaction in specific prioritized activities, and broader changes in personal recovery, autonomy, or experience of services are related but distinguishable processes. Person-centered care itself also extends beyond the existence of individualized goals. Santana et al. organize person-centered care implementation across three interdependent domains of structure, process, and outcomes and emphasize that the structural context of a service provides the foundation for person-centered processes and influences their outcomes (40).

This perspective is particularly useful for understanding the support model evaluated. The model incorporates elements that are clearly compatible with person-centered planning. Life history, needs, preferences, and personal goals are used to develop an individualized support plan, consistent with approaches in which valued life goals occupy a central place in recovery-oriented planning (11). Stanhope et al., for example, describe person-centered care planning as the collaborative co-creation of a recovery-oriented service plan based on the individual’s most valued life goals (11).

However, implementation of those individual plans occurs within standardized procedures and a predefined regional portfolio of programs, support intensities, and support modalities. This is not a characteristic attributable specifically to the collaborating organization, which operates within the framework established by the public system, but rather to the service architecture within which support is delivered. This distinction makes it useful to separate individualization of planning from individualization of the response that can subsequently be provided.

The first depends on enabling the person to identify needs, preferences, and goals and to participate in constructing the support plan. The second also depends on whether the service architecture has sufficient flexibility to respond to the diversity and evolution of those priorities. The implementation literature supports this distinction. Lodge et al. identified barriers at multiple levels to person-centered recovery planning within public mental health organizations, with organizational context playing an important role (41). In a systematic mixed-studies review of 70 studies, Piat et al. identified flexibility, relationship building, incorporation of lived experience, organizational and policy commitment, and the availability of resources to support personal recovery goals among the factors influencing implementation of recovery-oriented services (12). In Spain, Sampietro et al. similarly discuss opportunities and barriers to translating community-based, person-centered, rights-based recovery principles into public mental health policy and services (13).

A second issue concerns how outcomes are monitored within the service system. Although support characteristics are documented using standardized categories of intensity and modality, routine planning and follow-up are based primarily on life history, individualized interviewing, professional assessment, and the criteria established by the public system, without systematically integrating standardized outcome measures covering personal recovery, occupational performance and participation, experience of care, or goal attainment. This does not reduce the value of narrative and professional assessment, which is essential for understanding an individual’s history, preferences, and circumstances. Rather, it raises the question of how these approaches might be complemented.

Lewis and Killaspy note that standardized outcome measures reported by service users can be useful for monitoring individual progress, while also emphasizing that outcome and experience measures require careful interpretation when they are used to evaluate service quality (42). Santana et al. similarly include measurement and monitoring of person-centered care performance among the structural components needed to support person-centered care (40). Integrating narrative information with standardized outcome monitoring could therefore improve the ability to trace relationships among identified needs, personal goals, planned supports, supports actually received, and outcomes experienced by the person, while preserving individualized decision-making.

Within the rights-based framework outlined in the Background, Empowerment extends beyond the opportunity to express preferences and includes meaningful participation in decisions and in the ongoing review and adaptation of support related to one’s life goals.

### 4.6 Sex, diagnosis, and heterogeneity of support needs

The findings also underscore the importance of considering sex, diagnosis, and support characteristics together. The predominance of male participants should be interpreted in the context of the specific population served by the collaborating organization. More than half of the sample (54.1%) received social reintegration support for people with criminal records as their primary support program, and schizophrenia spectrum and other psychotic disorders were also proportionally more frequent among male participants. The sex distribution may therefore reflect, at least in part, the service’s specific program composition, referral pathways, and diagnostic profile. Accordingly, transferability of the findings is most directly applicable to comparable routine community support settings and populations with similar service characteristics.

The distribution of primary diagnostic groups differed between female and male participants, particularly for schizophrenia spectrum and other psychotic disorders and depressive and bipolar disorders. The literature on schizophrenia describes sex-related differences in clinical and psychosocial characteristics, including age at onset, premorbid functioning, negative and affective symptoms, and some comorbidities, while also documenting inconsistencies and substantial heterogeneity across studies (43). This supports interpreting sex and diagnosis jointly rather than treating either factor in isolation.

Despite differences in diagnostic composition, there was no clear evidence that the longitudinal pattern of the primary outcomes differed between female and male participants. This does not imply that sex is irrelevant to recovery; rather, it indicates that differential change by sex was not detected in this sample during the observed period. Exploratory analyses considering sex and diagnosis jointly likewise did not provide statistically significant evidence of different longitudinal trajectories. Lases et al. found that the five CHIME processes were represented across recovery narratives from different diagnostic groups (3), supporting a transdiagnostic perspective while recognizing that individual recovery trajectories remain shaped by clinical, social, and personal circumstances.

Primary diagnostic group was also associated with the configuration of support received, with different diagnostic groups more frequently receiving different support intensities and modalities. This illustrates that diagnostic heterogeneity is accompanied by heterogeneity in support needs and service responses. The adjusted differences observed in COPM performance and satisfaction between diagnostic groups should be interpreted within this context. They represent between-group differences averaged across assessment times and do not indicate that one diagnostic group improved more than another during the evaluation period. This distinction is consistent with evidence that personal recovery has only partial relationships with symptoms and clinical functioning (6) and with the transdiagnostic nature of CHIME processes (3).

Associations between support characteristics and outcomes require similar caution. In a system in which support intensity, modality, and combinations of services are assigned according to individual support needs, people receiving different configurations of support are unlikely to constitute naturally equivalent groups. Such associations are therefore more informative for characterizing heterogeneity in the population and the organization of support than for establishing the superiority of a particular support modality or intensity. This perspective is consistent with the relevance of social and contextual influences on mental health recovery (37) and with evidence that community models must be capable of responding to heterogeneous needs and preferences (14).

### 4.7 Implications for community mental health services

The findings have implications for the evaluation and development of community mental health services. Their value does not lie in suggesting that one outcome measure is superior to another. Rather, personal recovery, occupational performance and satisfaction in activities prioritized from problems in occupational performance or participation, and the perceived experience of person-centered care address different questions and provide complementary information. Assessing these levels together can provide a more differentiated account of change among people receiving community support, consistent with evidence distinguishing functional, social, and existential recovery outcomes (5) and with recent research emphasizing multiple recovery outcomes and the centrality of service-user goals in community models of care (14).

From a public mental health perspective, evaluation of community services should extend beyond documenting which services are provided and at what level of intensity. It is also relevant to understand the needs and goals those services are intended to address, the person’s involvement in defining them, and the outcomes experienced in areas of life that matter to that person. Implementation evidence further indicates that recovery orientation depends not only on professional practice but also on organizational conditions, flexibility, resources, and the capacity to support diverse personal recovery goals (12).

The present findings particularly emphasize continuity across three components of service delivery: facilitating the identification and development of priorities with the person, translating those priorities into concrete supports, and evaluating outcomes from the person’s perspective. The heterogeneity in diagnostic profiles and support configurations observed in this study reinforces the need for community systems that can combine individualized planning with mechanisms for reviewing whether supports remain responsive to changing needs, occupational performance and participation problems, and life goals (11, 12, 34, 40, 41).

Combining life-history information and professional assessment with appropriately selected standardized outcome measures may support this process without replacing the relational and individualized nature of person-centered planning. The value of standardized measures in this context is to provide complementary longitudinal information that can help determine whether support remains aligned with identified priorities and with changes experienced by the person. Evaluation can thereby function as part of the ongoing individualization and quality improvement of community support rather than solely as a research or accountability exercise.

International longitudinal community-service studies provide useful, although not directly equivalent, comparators. In a rural South Australian Community Mental Health Rehabilitation Service, Leet et al. observed significant increases in RAS-DS scores between admission and discharge, although the sample was small and the service was residential (44). By contrast, Owusu et al. reported no significant change in RAS scores over 6 weeks among participants receiving routine follow-up after discharge from acute mental health care in Alberta, Canada (45). In Hong Kong, Tse et al. found that Recovery Assessment Scale scores in participants receiving Strengths Model Case Management were broadly stable over 6 and 12 months, with no significant between-group improvement in most outcomes compared with usual community care (46). These studies illustrate that longitudinal recovery findings in community mental health settings are heterogeneous and sensitive to service model, population, follow-up interval, and outcome measure. The stable RAS-24 finding in the present study therefore falls within a broader international pattern in which personal recovery does not necessarily show uniform short-term change, while direct effect-size comparisons remain inappropriate because the designs and service contexts differ.

## 5 Limitations

Several limitations should be considered when interpreting these findings. First, the pre–post design without a comparison group limits causal inference. Although withholding or delaying routine support for research purposes was not considered appropriate in this service context, alternative observational or quasi-experimental studies were not part of the original evaluation design and could strengthen causal inference in future studies. Such designs could help distinguish changes occurring during routine support from temporal, contextual, or other concurrent influences. Contextual factors, concurrent experiences or services, natural variation over time, or other unmeasured influences may also have contributed to changes between assessments. Participants were not necessarily new to community support at baseline. The evaluation coincided with the implementation of an updated regional framework, and for some participants this involved the continuation, modification, or reorganization of supports already being received rather than the initiation of an entirely new service. Baseline measurements should therefore be interpreted as assessments conducted before the predefined study evaluation period, rather than as measurements obtained before first exposure to community support. Detailed participant-level data on contact duration and setting, the number of contacts beyond the minimum weekly contact, and the timing of individualized plan reviews were not systematically available for the present analysis. Similarly, although the updated framework could involve continuation, modification, or reorganization of existing supports, these within-period changes were not quantified systematically. This limits component- and dose-specific interpretation of the support received but does not alter the study’s primary focus on longitudinal participant-reported outcomes during routine community support. The findings should therefore be interpreted as longitudinal changes observed during the period of community support rather than as causal effects of the support provided. At the same time, conducting the study under routine service conditions strengthens its relevance to real-world community practice.

Second, assessments were conducted within study-wide assessment windows rather than at an identical participant-specific interval. Assessing all 159 participants using the study battery required baseline and follow-up assessments to be distributed over several weeks. To maximize temporal comparability, follow-up assessments were scheduled following the same order in which baseline assessments had been completed. Nevertheless, the individual interval between baseline and follow-up was not exactly identical for all participants. In addition, community support continued beyond the research evaluation period as part of routine service provision. The study therefore characterizes changes during the evaluated period but does not establish whether the observed pattern was maintained over the longer term. Response-style tendencies, such as preferential use of midpoint or extreme response categories on the fixed-item questionnaires, were not independently assessed; therefore, their potential influence on RAS-24 and CCRQ-e scores cannot be excluded. In addition, because the quantitative outcome measures were administered in a fixed order and fatigue was not formally assessed, potential order or fatigue effects cannot be completely excluded.

Third, outcome availability differed across measures. The Family Involvement dimension of the CCRQ-e was optional and its applicability depended on each participant’s family circumstances, which reduced data availability for the 7-dimension score, particularly in analyses requiring observations at both assessment points. Lower availability for this dimension may therefore reflect, at least in part, substantive differences in participants’ family circumstances rather than nonspecific missingness alone. However, specific reasons for non-completion were not systematically coded for analysis. The sensitivity analysis using the 6-dimension score excluding Family Involvement yielded the same substantive conclusion, indicating that the overall CCRQ-e finding did not depend on inclusion of this dimension. Missing values were not imputed, and the mixed models used all available observations.

Fourth, the individualized nature of the COPM has implications for group-level interpretation. Participant-level performance and satisfaction scores were calculated from all valid priorities identified by each person, but both the number and content of those priorities differed across participants. Similar participant-level mean scores may therefore summarize different numbers and types of occupational performance or participation problems. This individualization is an essential feature of the COPM and is particularly consistent with a person-centered approach, but it limits direct comparability of the content underlying aggregated scores. The present quantitative analysis did not examine the specific content of the priorities identified by participants or include variables designed to assess factors associated with the number of priorities identified. Consequently, it was not possible within the scope of this analysis to determine which specific areas of occupational performance or participation contributed most to the observed changes or to quantitatively explain why some participants identified fewer priorities. Examining the content and development of these priorities in greater depth represents an important direction for future research.

Fifth, the unequal distribution of female and male participants reduced precision for detecting sex-related differences, particularly in the more complex exploratory interactions simultaneously involving sex and diagnostic group. Only sex, as recorded in organizational records, was available for analysis; gender identity was not collected. Consequently, potential differences associated with gender identity could not be examined. The absence of statistically significant interactions should therefore not be interpreted as evidence of equivalence between female and male participants. Primary diagnoses also had to be grouped for adjusted analyses because some individual diagnostic categories were too small to provide sufficiently stable estimates. In particular, depressive and bipolar disorders were combined to obtain an analytically viable group. Although this approach improved statistical feasibility, these are clinically distinct conditions, and grouping them may have obscured diagnosis-specific patterns. Specific primary and co-occurring diagnoses were therefore retained in the descriptive analyses.

Associations involving diagnosis, support characteristics, and outcomes should also be interpreted in accordance with the service context of the study. Support intensity, modality, and combinations of support were not experimental exposures but components of the response provided by the service system according to each person’s identified support needs. Individuals receiving different support configurations would therefore be expected to differ in their underlying support needs. Although the adjusted models accounted for several demographic, diagnostic, and service-related characteristics, associations between support characteristics and outcomes should not be interpreted as causal effects of a particular support intensity or modality. Their primary value lies in characterizing heterogeneity in support needs and service responses within the population studied.

CHIME was used as a conceptual framework to guide the selection, evaluation, and interpretation of outcomes rather than as a scoring system intended to provide independent quantitative measures of each of its five processes. The selected instruments provide complementary information conceptually related to CHIME: the RAS-24 assesses personal recovery and includes content related to all five processes, although with unequal representation; the COPM provides an individualized assessment particularly relevant to Meaning through the identification of occupational performance and participation problems and subsequent ratings of performance and satisfaction; and the CCRQ-e captures the perceived experience of person-centered care. Accordingly, interpretation through Connectedness, Hope, Identity, Meaning, and Empowerment should be understood as a theoretically informed integration of complementary outcomes rather than as independent psychometric measurement of each CHIME process. This limits direct quantitative comparison across the five processes but is consistent with the conceptual purpose of the study.

Finally, the study was conducted through a single collaborating organization operating within one regional public social services system. Regulatory, organizational, and service-delivery characteristics may differ across regions and countries, which should be considered when assessing the transferability of some service-level implications. The program evaluated was part of routine care and was defined and delivered by the public system and the collaborating organization independently of the research team; the research therefore did not design or modify the intervention. Although the present quantitative study forms part of a broader mixed-methods project that also includes qualitative exploration of participants’ experiences and the meanings they attribute to them, people with lived experience did not formally participate as research partners in decisions concerning the study design or selection of quantitative outcomes. Future evaluations could complement the direct inclusion of participants’ experiences with greater involvement of people with lived experience in research design and outcome selection, as such involvement may enhance the relevance and acceptability of the outcomes assessed (47).

## 6 Conclusion

This CHIME-informed evaluation of routine community mental health support identified a differentiated pattern of longitudinal change. No uniform pattern of improvement was observed across the complementary recovery-related outcomes. During the evaluation period, statistically significant mean increases were observed in occupational performance and satisfaction with performance in activities prioritized by participants on the basis of occupational performance or participation problems; however, the adjusted mean changes were less than one point on the 10-point COPM scales. No statistically detectable longitudinal change was observed in overall personal recovery or perceived person-centered care. No clear evidence was identified that longitudinal changes in the primary outcomes differed between female and male participants.

Viewed through the CHIME framework, these findings indicate that different components relevant to recovery may follow different trajectories during the same period of community support. The findings therefore support considering personal recovery, occupational performance and participation problems, performance and satisfaction in prioritized activities, and service experience as complementary rather than interchangeable components when evaluating community mental health support.

The findings also have implications for service evaluation. The model studied incorporates individualized planning based on each person’s life history, needs, preferences, and personal goals, from which the service system organizes the available supports. Complementing this process with individualized and standardized outcome measures such as those used in the present study may make changes in recovery-related outcomes more visible than documentation of support provision alone. Integrating this information with life-history-based planning and professional assessment could support more systematic monitoring of the relationship between person-defined priorities, supports provided, and outcomes experienced, without replacing the individualized and relational nature of person-centered planning.

From a public mental health perspective, such an approach may strengthen continuity between planning, support provision, and outcome evaluation and provide useful information for reviewing and adapting community services to heterogeneous and changing support needs and life goals. Because of the pre–post design without a comparison group, the observed changes should not be interpreted as evidence of causal program effectiveness. Rather, the study provides real-world evidence that different recovery-related outcomes may evolve differently during routine community support and that multidimensional, person-centered evaluation can provide additional information for monitoring and developing community mental health services.

## Data Availability

The datasets generated and analyzed during the current study are not publicly available due to ethical and confidentiality restrictions but are available from the corresponding author on reasonable request.
